# Whether to Save a Robot or a Human: On the Ethical and Legal Limits of Protections for Robots

**DOI:** 10.3389/frobt.2021.712427

**Published:** 2021-07-07

**Authors:** Kamil Mamak

**Affiliations:** Department of Criminal Law, Jagiellonian University, Kraków, Poland

**Keywords:** moral patiency, moral circle, robot rights, moral dilemma, trolley problem

## Abstract

Proponents of welcoming robots into the moral circle have presented various approaches to moral patiency under which determining the moral status of robots seems possible. However, even if we recognize robots as having moral standing, how should we situate them in the hierarchy of values? In particular, who should be sacrificed in a moral dilemma–a human or a robot? This paper answers this question with reference to the most popular approaches to moral patiency. However, the conclusions of a survey on moral patiency do not consider another important factor, namely the law. For now, the hierarchy of values is set by law, and we must take that law into consideration when making decisions. I demonstrate that current legal systems prioritize human beings and even force the active protection of humans. Recent studies have suggested that people would hesitate to sacrifice robots in order to save humans, yet doing so could be a crime. This hesitancy is associated with the anthropomorphization of robots, which are becoming more human-like. Robots’ increasing similarity to humans could therefore lead to the endangerment of humans and the criminal responsibility of others. I propose two recommendations in terms of robot design to ensure the supremacy of human life over that of humanoid robots.

## Introduction

Robots are increasingly entering the social lives of humans, which raises certain questions about our mutual interaction, such as whether robots are mere tools or something more, how we should treat robots, whether we owe robots anything, and whether robots should have rights. In recent years, increased academic attention has been paid to such issues, and many important publications have been published on these themes (cf. Balkin 2015; Darling 2016; Gunkel 2018b; Pietrzykowski 2018; Turner 2018; Abbott 2020; Bennett and Daly 2020; Gellers 2020; Nyholm 2020; Smith 2021). Schröder stated that “controversies about the moral and legal status of robots and of humanoid robots in particular are among the top debates in recent practical philosophy and legal theory” (Schröder 2020, 191). The discussion of robots’ possession of rights is strongly connected with deliberation on their moral status, another of the principal topics considered in the ethics of artificial intelligence (Gordon and Nyholm 2021). A few review works concerning such issues have recently been published (Schröder 2020; Gordon and Pasvenskiene 2021; Harris and Anthis 2021).

In this paper, I focus on the limits of the protection of robots by answering the question of who should be saved–human or robot. Some people have indicated that they would hesitate to sacrifice robots to save humans. Nielsen et al. examined how the anthropomorphization of robots impacts the decisions of humans in a moral dilemma when there is a need to sacrifice one entity to save another. The authors’ results indicate that “when people attribute affective capacities to robots, they become less likely to sacrifice this robot to save a group of human beings” (Nijssen et al., 2019, 53). These results are alarming from the perspectives of both ethics and law. Current legal systems take the stance that human life is at the top of protected values. Furthermore, not saving humans in a situation in which there is the possibility of doing so could be considered a crime.

As robots are becoming increasingly human-like, this issue will continue to gain importance over time. The following question thus emerges: Should we act in order to maintain human life as the most valuable from the legal perspective? For example, if we accept that human life should always be at the top of hierarchies of value, perhaps manufacturers should be forced to mark robots such that they can be easily differentiated from humans in emergencies. In unforeseen traffic accidents, drivers only have seconds to decide what to do and what they can avoid. Robot drivers and human drivers should know that robots should be sacrificed in collisions involving both humans and robots. From another perspective, we should ask whether robots have any properties that make them equal to humans with regard to legal protections, such as a human-like intelligence, and whether we could in fact decide that robots should be granted more protection than humans. I respond to all of these issues in this paper, which is structured as follows.

I start by considering the issue of rights for robots and presenting popular ways of ascribing moral patiency to robots. I then explore conflict situations between the lives of robots and those of humans on the basis of the presented approaches. The subsequent section is devoted to the contemporary hierarchy of values set by law; here, I demonstrate that a person who hesitates to sacrifice a robot could be considered to have committed a crime. Finally, I offer recommendations for modifying the design of robots to mitigate the described risks and present the conclusion of this study.

## Rights for robots?

Could a robot have rights? The short answer to this question is “yes”. Law is a social technology (Fairfield 2021), and we can, in theory, do whatever we want with it. According to a popular anecdote, Caligula made his beloved horse Incitatus a consul. Whether true or not (and it seems not; Barrett 2015, 289), this anecdote illustrates that someone who has the power to create the law can theoretically do almost whatever they want. The law is a flexible tool, and if there is a need, it can be used for different purposes. Gellers, for example, noted that ships had formal legal status in history because there was such a need (Gellers 2020). Hence, there is no theoretical obstacle to granting rights to robots.

More demanding is the “should” question, which is tied to the issue of the moral standing of robots. If robots were welcomed into the moral circle, we could expect that human interactions with robots would be impacted by their possession of moral status. Some scholars categorically argue that robots should not be granted moral status (cf. Bryson 2010; Birhane and van Dijk 2020), but there is also a significant body of literature that claims otherwise. I briefly present four approaches to determining the moral patiency of robots: properties-based, indirect duties, relational ethics, and environmental ethics.

The most widely accepted approach to granting moral status to robots is based on what a robot “is”. To decide whether an entity is qualified to enter the moral circle, we must know its ontology. If that ontology contains the qualities that we believe are important, we accept that the entity is in the moral circle. In some approaches, a quality or set of qualities is sufficient to resolve the moral status of robots. Other approaches discuss properties such as sentience, intelligence, or consciousness (cf. Floridi and Sanders 2004; Sparrow 2004; Himma 2009; Levy 2009; Hildt 2019; Kingwell 2020; Mosakas 2020; Gibert and Martin 2021; Véliz 2021). Thus, if a robot can feel pain or is self-aware, then we should incorporate it into the group of entities that possess moral status. An approach based on properties seems a useful tool by which to grant moral status in theory, but in reality presents several issues. First, there is no consensus as to which quality/qualities should be sufficient for moral consideration, as different authors have identified different qualities on which to ground moral patiency. Second, there is no consensus as to what human qualities are; we still do not know what it means to be conscious, self-aware, or intelligent (cf. Umbrello and Sorgner 2019). Third, as Gunkel wrote, the basis of moral status on qualities serves as a way of postponing the discussion (Gunkel 2018b). Fourth, as Coeckelbergh observed, there are epistemological limitations (Coeckelbergh 2010, 212), such as how to know whether a robot is feeling pain (cf. Dennett 1978; Bishop 2009; Adamo 2016). We already struggle to determine the inner states of other human beings; robots could be much harder to “read”.

Danaher proposed an interesting response to the epistemological problem through the theory of ethical behaviorism, “[…] which holds that robots can have significant moral status if they are roughly performatively equivalent to other entities that have significant moral status” (Danaher 2020, 2023). Danaher did not focus on what robots are, but rather on how they perform in everyday life (i.e., the observable aspect of their functioning). If robots cross the performative threshold of entities that have moral status, we should treat them as such entities. Some scholars have criticized ethical behaviorism (Nyholm 2020; Smids 2020), and I describe one issue created by this theory in a later section. However, ethical behaviorism is the most practical response to the lack of knowledge concerning the qualities of entities with which we are interacting–if we believe that qualities matter.

The second popular approach to moral patiency is grounded on the Kantian theory of indirect duties toward animals. Kant believed that animals do not have (direct) moral status, but that humans should treat them well regardless. He claimed that

if a man has his dog shot, because it can no longer earn a living for him, he is by no means in breach of any duty to the dog, since the latter is incapable of judgment, but he damages the kindly and humane qualities in himself, which he ought to exercise in virtue of his duties to mankind. Lest he extinguish such qualities, he must already practice a similar kindliness toward animals; for a person who already displays such cruelty to animals is also no less hardened toward men. (Kant 1997, 212).

Proponents of this theory liken robots to animals in order to advocate for granting moral status to robots and thereby preserving our own humanity. One of the proponents of this approach is Kate Darling, who developed the analogy of robots to animals (Darling 2016). Darling suggested that robots are new animals and that we should consider how humans previously resolved issues in our relationships with animals to prepare for our existence alongside robots (Darling 2021). Smith also developed a Kantian approach, which advocates treating robots as moral patients to prevent their dehumanizing use and to protect the dignity of humans (Smith 2021). Coeckelbergh connected this approach to the relational turn, which I briefly discuss below (Coeckelbergh 2020b).

The relational turn in roboethics is largely associated with two authors, Coeckelbergh and Gunkel, who did not limit their deliberations to robots (cf. Coeckelbergh and Gunkel 2014). In their view, the moral patiency of robots is not grounded on robots’ ontological properties, but, crucially, on the relations between robots and humans (Coeckelbergh 2010). In this approach, ethics precedes ontology and is usually the opposite (Gunkel 2018b). As Gunkel noted, “[…] the question of social and moral status does not necessarily depend on what the other is in its essence but on how she/he/it (and the pronoun that comes to be deployed in this situation is not immaterial) supervenes before us and how we decide, in “the face of the other” (to use Levinasian terminology), to respond” (Gunkel 2018a, 96). Coeckelbergh claimed that “we could argue that, […], the status of AIs will be ascribed by human beings and will depend on how they will be embedded in our social life, in language, and in human culture” (Coeckelbergh 2020a, 59).

The last approach that I want to mention here is environmental ethics. This approach is neither fully distinct from the concepts presented earlier (e.g., the approach based on a Kantian view of animals) nor a homogeneous concept. Different strands in environmental ethics differ in their response to questions concerning how humans should relate to the environment and non-human entities and how to situate humans among them (cf. Brennan and Lo 2021). However, in his book on rights for robots, Gellers embedded the issue of robots within the concept of environmental ethics, suggesting that determining the moral standing of robots could be a “side effect” of discussion of the moral status of nature and its elements. Gellers advocated for a critical environmental ethics approach according to which the idea of recognition of robots’ rights (and those of other non-natural entities) is related to epistemic pluralism (Gellers 2020). This approach may, for example, be focused on the harmony between the elements of nature, with one such element being technological artifacts, including robots. If we grant moral standing to trees (cf. Stone 2010), why not to robots? This environmental approach is also supported by the religious beliefs of non-Western cultures, which are discussed in depth in Gellers’ book.

## Robots’ Right to Life

Moral standing may be granted to robots on many different grounds. Possessing moral consideration is the basis for possessing rights (Danaher 2020). However, the question remains which rights are to be possessed. Accepting the notion that it is possible for robots to possess rights says little about the content of such rights; this is another issue that requires further deliberation (cf. Graaf et al., 2021). Humans enjoy various types of rights, ranging from the right to privacy to the right to free expression and the right to holidays. Some of these rights are transferable to robots, while others are not. Furthermore, robots could potentially have specific rights resulting from their distinct ontology. This paper, however, is not the appropriate place to expand on this issue; instead, I limit my deliberations to the concept of what could be called the “right to life.”

The right to life is one of the basic rights that might be derived from the acceptance of robots’ moral standing; here, I use “might,” because it is not obvious (see Lima et al., 2020, 135:6). I would like to note two objections: First, assessing whether robots exist or not alone is problematic. Indeed, such determinations are problematic for humans as well. The criteria used to determine death are legally and ethically unclear and have changed during the course of history, for example, from the irreversible loss of heart and lung function to the death of the brain (cf. Belkin 2014; De Georgia and Michael, 2014). There are still occasional protests concerning whether we should turn off life-support apparatuses, even in cases where brain death has been confirmed. Assessing whether a robot no longer exists could be even more problematic. Is a robot “dead” when all of its data are stored online, but the physical body is destroyed? Second, it is not clear that the right to existence is a basic right. If we use the example of animals, we could say that some animals are in the moral circle in terms of animal rights; still, it is possible to kill even these animals for certain purposes, such as for food or clothes. From a legal perspective, it is possible for a farmer who breeds animals to kill them legally and to be punished for cruelty to the very same animals he kills. For the purpose of further deliberation, I ground the notion of the right to life in the meaning that any breach of that right will destroy a robot completely.

What should be noted is that the deliberations on a robot’s “right to life” do not mean that “robot rights” are some kind of an extension of human rights. In this particular instance there is just a similarity to the concept of the right to life (which itself belongs to the domain of human rights). The set of possible robot rights is different from the set of human rights (cf. Gunkel 2020).

Different elements of our social and biological world-such as e.g., corporations, animals and nature-have already been determined to possess certain rights in different places around the word (see more on that Gellers 2020). The discussions about particular rights of [certain kinds of] robots should be treated similarly–that is: as discussions on rights of non-humans. What is more, robots (like pets or farm animals) are someone’s property, and this characteristic makes them legal objects and not legal subjects. Legal subjects are legal persons - both natural and artificial (i.e., corporations)-and legal personhood is associated with a wider scope of legal rights (more on the concept of legal personhood: Kurki 2019). Having in mind this division into legal objects and subjects the treatment of robot rights as an extension of human rights seems even more inaccurate.

It is one issue to claim that robots have moral standing; how we situate robots in the hierarchy of values is another issue. Now I turn to how best to resolve the dilemma of whether the lives of robots should be more or less valued than the lives of humans, or, in other words, who we should save according to the previously presented approaches.

Let us assume that properties such as sentience and intelligence are not binary concepts, but a spectrum. We could, on that ground, say that different entities are situated at different points on such scales. In his book *Superintelligence*, Bostrom situated different organisms in this way regarding their intelligence (Bostrom 2016). Simpler organisms are lower on the scale, while human beings are at the top. This thinking allows us to assert that, for now, human beings are at the top of the scale, which justifies their privileged position over other inhabitants of our planet. However, what if robots were to exceed humans in terms of those properties that we believe provide the basis for moral standing? Should we recognize their superiority over us and, for example, prioritize them in a dilemmatic situation? My deliberations here are extremely speculative due to the plurality of philosophical concepts involved and the problem of epistemological limitations. However, a question like this could arise at some point, especially in the context of the priorities that the law assigns to human beings. We must think about how we want to organize our world with regard to entities that are situated at different points on the scale in relation to human beings.

There are three potential answers to the question about prioritization. If robots possess qualities that correspond to qualities of entities that are lower on the hierarchy of values (e.g., robots with insect-like intelligence), we should prioritize humans. A more complicated answer results in the case of entities that are, more or less, the same as humans. Bearing in mind how difficult it may prove to determine what is “like” a human, we can imagine that robots may be like us. In this scenario, it seems appropriate that we should treat robots as equal to human beings. In (Putman, 1964) observed that the materials used in the construction of a robot should not matter; what should matter is the qualities the robot possesses (1964). Prioritizing human beings could be seen as discrimination based on the materials used to build an entity. In this thinking, the question of prioritization is unanswerable; it would be similar to asking whether we should prefer older people to younger people or men to women. Such an *a priori* decision could be seen as discrimination and thus be forbidden by law. The most controversial answer would result if robots outperform humans in the qualities that we consider to be a source of moral standing. It cannot be excluded that priority should b given to robots, and Sparrow defends such a position (Sparrow 2004).

The approach based on Kantian indirect duties is the easiest means to answer the prioritization problem. Kant believed that animals do not have moral status; therefore, robots also do not have (direct) moral status. Humans, however, do have such status. Thus, in conflict situations, we should save human beings.

In contrast, the relational approach is the most unclear in regards to resolving the prioritization problem. This approach is focused on the relations of human beings with robots, not on robots’ ontology. On the one hand, the relational approach says little about how to deal with a conflict situation. On the other hand, this approach is, in a sense, anthropocentric. The relations that ground moral standing originate from humans; human relations are the starting point for ethical decisions. From that perspective, human beings will take precedence over any other entities with whom humans have relations. Gunkel adopted the relational view proposed by Levinas on the grounds of roboethics and also admitted that Levinas made an anthropocentric interpretation of his own works (Gunkel 2018a, 97). However, during the recent workshop “Rabbits & Robots: Debating the Rights of Animals & Artificial Intelligences” organized by the Cambridge Centre for Animal Rights Law (Cambridge Centre for Animal Rights Law 2021), Coeckelbergh suggested that the relational approach is anthropocentric, but epistemically, and not necessarily morally. Nevertheless, even taking this clarification into account, it is still unclear whether it is permissible to sacrifice humans to save robots.

Environmental ethics is not homogenous, and there are different possible answers under this approach to the prioritization question. In the anthropocentric view of the environment, priority is given to human beings. The modern version of anthropocentrism is called “enlightened anthropocentrism” or “prudential anthropocentrism” (Brennan and Lo 2021). These views regarding the environment are also connected with the Kantian view presented above and similarly resolve the conflict situation, namely by answering that human beings should be saved. According to anthropocentric environmental ethics, there is no priority granted to robots as non-human entities, nor to other non-humans. However, it is also possible to arrive at the opposite answer on the grounds of non-anthropocentric environmental ethics, such as bio-or ecocentrism. For example, in eccentric environmental ethics, humans do not enjoy priority over other species, as the ecosystem is considered as a whole. Describing the deep ecology movement, which could be seen as ecocentric, Naess stated that it rejects “the man-in-environment image in favour of the relational, total-field image” (Naess 1973, 95). The non-anthropocentric view was also advocated by Gellers (Gellers 2020). Gellers’ critical environmental ethic is ecocentric and holist, positing that all vulnerable entities present in an open ecology are radically equal. His approach takes inspiration from non-Western and indigenous worldviews. In the context of non-anthropocentric approaches, it is worth mentioning a case from 2016, when a Cincinnati Zoo worker killed a gorilla to protect a three-year-old who had fallen into the gorilla’s enclosure (Panagiotarakou 2016). In that case, environmental ethicists were not certain that the zookeeper should have killed the gorilla (cf. Bein and McRae 2020), indicating that this perspective is open to the possibility that non-human beings have priority. If the destruction or killing of non-human entities would do irreparable damage to nature and the harmony between its elements, then a human being could be sacrificed.

In sum, who should we save, the human or the robot? The answer is most ambiguous under the properties-based approach. In some scenarios, the properties-based approach indicates that the priority should be given to humans, but, in others, prioritizing humans could be considered an act of discrimination. It is even possible to imagine that priority should be given to robots if their qualities outperform those that we believe to be the basis of moral standing. In an approach based on Kantian indirect duties, the answer is clearer: We should save human beings, as they are the entities with direct moral status. In the relational approach, the priority is also (probably) given to humans as the source of the relations. Finally, on the basis of environmental ethics, the answer depends on the initial starting point. The anthropocentric approach prioritizes human beings, but the answer is more unclear in relation to non-anthropocentric views, according to which preference may be given, in some cases, to non-human lives.

Although it is beyond this paper’s scope, which is dedicated to the conflict between humans and robots, another intriguing version of the prioritization problem could arise when we raise similar questions in the context of a dilemma involving animals and robots (cf. Wilks et al., 2021). There is already a growing body of literature looking at the interactions among animals and robots (cf. Butail et al., 2014; Romano et al., 2019).

## Saving Robots Instead of Humans is a Crime

Previous deliberations concerning ethics have been normative in nature, focusing on how humans should behave and starting with different ethical assumptions. These deliberations are useful for discussing how humans should organize our mutual life with robots in the future. All of the previously introduced approaches are theoretically possible to adopt, with some obstacles. Indeed, some of them are already part of the social order, such as non-anthropocentric environmental approaches in some Native American tribal lands (see Gellers 2020). However, it is difficult to imagine that these approaches would be easily universalized for translation from one jurisdiction to the next, for example, into Western systems.

The current answer to the question of who should be sacrificed between humans and robots is connected to the hierarchy of values embedded in legal systems. Hesitation to sacrifice robots in order to save humans, as exhibited in the research of Nijssen et al. (2019), is highly problematic from the perspective of contemporary law, and such behavior could even be a crime. The remainder of this section focuses on this issue.

The law is human-centered, and in case of dilemmas–human life vs. non-human life, there is almost no doubt that human life is favored. The right to life and physical security is the most basic claim of every human being (Ashworth 1975, 282). According to the modern understanding of human rights, the individual human being is put in the center as the goal and the end, and the right to life is a fundamental human right (Ziebertz and Zaccaria 2019b). There is a legal obligation to protect life, and any exemptions are highly controversial, such as abortion, killing in self-defense, euthanasia, and the death penalty (see on those issues, cf. Ziebertz and Zaccaria 2019a; Fletcher, 1978). Even a cursory legal assessment reveals that the right to life of a human being is highly protected at the international, regional, and national levels. Many laws declare humans’ “right to life,” often citing the United Nations Universal Declaration of Human Rights from 1948, which states in Article three that “everyone has the right to life, liberty and security of person”. For a prioritization dilemma, a second document is even more informative: The European Convention on Human Rights (ECHR). According to Article 2,

1. Everyone’s right to life shall be protected by law. No one shall be deprived of his life intentionally save in the execution of a sentence of a court following his conviction of a crime for which this penalty is provided by law.
2. Deprivation of life shall not be regarded as inflicted in contravention of this Article when it results from the use of force which is no more than absolutely necessary: 1) in defence of any person from unlawful violence; 2) in order to effect a lawful arrest or to prevent the escape of a person lawfully detained; 3) in action lawfully.

According to this provision, there is no possibility of deprivation of human life in order to save non-human entities. Clause (a) of Article 2.2 states that deprivation of life is allowed under some circumstances if it is necessary to protect the life of another human being.

People should not only not take human life, but sometimes are obliged–under a threat of punishment–to actively protect human life. I now briefly discuss this obligation. Criminal law is one of the branches of law most resistant to harmonization, and some important features of criminal responsibility are not derived solely from a single provision. I illustrate the resulting problems using an example from a specific real-world system, namely that of Poland. Polish criminal law includes a crime called “failure to render aid,” which is useful to examine the problem of hesitation to sacrifice robots to save humans. As stated in the Polish Criminal Code, Article 162, this crime is defined as follows:

§ 1. Whoever does not provide assistance to a person being in an immediate danger of loss of life or sustaining a grievous bodily harm, even though he could have provided it without exposing himself or another person to a danger of loss of life or a danger of sustaining a grievous bodily harm, is subject to the penalty of deprivation of liberty for up to 3 years.
§ 2. Whoever does not provide assistance that requires a medical procedure, or in a situation where a prompt assistance can be provided by an institution or a person responsible for providing such assistance, does not commit a crime. (Wróbel et al., 2014).

Failure to render aid is a specific type of crime. Crimes usually concern behaviors that are not permitted, such as theft, murder, and rape. However, the legal system can also punish individuals for not doing something that it believes to be desirable. The system literally forces people to do something and, if they do not, threatens punishment. Because punishment for not doing something is an unusual case, it is used only in a limited set of examples, such as to regulate the actions taken when another human being is in a life-threatening situation. The legal system takes the view that if another human is in danger, a bystander cannot just look on, but must take action to help. The criminalization of failure to render aid is justified by one of the most commonly accepted moral norms–the need to help a person whose life or health is seriously endangered (Zoll [in:] Wróbel and Zoll 2017).

A few issues connected with the crime of failure to render aid require explanation. The first is obvious: The agent who can expect help is a human being rather than any other entity, animal, or non-living artifact. Only failure to help human beings is punishable by law. The law does not oblige people–under threat of punishment – to actively save animals, trees, or artifacts if there is a threat to their existence, even if they have high material or cultural value. Simply put, it is not a crime to watch and not help when an animal is dying or a tree is falling. One might regard such an act as morally corrupt, but it does not constitute a crime.

The second issue requires more explanation and is connected with the clause in the provision that reads “without exposing himself or another person to a danger of loss of life or a danger of sustaining a grievous bodily harm.” One could regard this clause as confirmation that the law does not require heroism: The obligation to act has limitations, and individuals are allowed to do nothing if there is a serious threat to themselves or to another human being. The crucial issue from the perspective of this paper is that the excuse not to help–and not to be liable for a crime–concerns the state of danger in which a human being, not an animal or any other artifact, finds themself. Individuals are obliged to do everything possible, including “sacrificing” non-human entities. Considering an illustrative example is helpful here. We can imagine that there is a person who is on fire, and a witness is wearing an expensive coat that could be used as a rescue tool. Nothing else nearby could be similarly used. The witness is obliged to use that coat to rescue the other person, even if doing so means that the coat will be destroyed. A dynamic situation such as this requires immediate reaction, which means the witness is obliged to react. If the witness does not act, he or she is committing a crime. The witness is obliged to act in the same way in terms of sacrificing animals and robots. In the case noted above of the gorilla at the Cincinnati Zoo, the zookeeper behaved correctly by saving the human child and killing the animal. The legal system is quite straightforward on this matter: The human being has a greater value, and if the zookeeper had not done what he did, he could be criminally liable for failure to render aid. Similarly, in a conflict between human life and robot “life”, failure to sacrifice the robot would constitute a crime.

The important issue as concerns the crime of failure to render aid–which results not from the description of the crime, but from the general rules of criminal responsibility–is intent to commit it (Wróbel and Zoll 2014). In the common law criminal literature, intent is associated with the concept of *mens rea* (cf. Lewna 2018; Zontek 2018)*.* Criminal intent means, in part, that the perpetrator is aware of all elements of a crime. In the case described in this paper, it means that a witness must be aware that another person is in a life-threatening situation and that they–or any other human–will not be threatened by providing help. For example, no crime will be committed if a person is lying on a bench in a park having a heart attack and needs medical intervention if the witnesses are not aware that the person needs help. A further example would be a witness who observes a child who is drowning. The witness knows that the child will die without help, but the witness cannot swim and is afraid that he or she will also die if he or she gives help. The witness is not aware that the water is 1 m deep, and there is no real threat. In this situation, no crime will be committed if the witness thinks that helping exposes him or herself to danger. According to criminal law, it is important what a person is thinking during the act under evaluation. If someone or something is deceiving a person, it will be considered. If, for example, a person thinks that he or she is interacting with humans, but is really interacting with robots (or vice versa), it could be crucial for determining criminal responsibility. If a person attacks a robot, thinking that they are attacking a human, that person could still be sentenced for a criminal attempt to attack a human, even if there was no human involved.

Hence, if a robot resembles a human and a person thinks that the robot is human and that not helping another human will save that human-like robot from danger, the person does not commit a crime. This, too, is an important notion. Lack of criminal responsibility does not mean that the situation is without difficult legal implications. The law can fail to achieve the goal of protecting humans in danger, which reveals the practical issue with Danaher’s ethical behaviorism. Danaher wrote about moral rights, not legal ones. However, in implementing his position within the scope of the law, a problem emerges. Danaher proposed the “the rule of actions,” which holds that we should treat robots like the entities they mimic (having in mind humans and animals); thus, if the entity resembles a human, we should treat it like a human. In this text, Danaher referred to the concept of the so-called philosophical zombie (cf. Kirk 2021) and argued that we should treat such entities as humans (Danaher 2020, 2029). The problem is not an objection to Danaher’s argumentation, which is coherent, but it demonstrates that this kind of thinking could have consequences that may be contradicted by the legal system, reflecting the gradation of values that places human life, over the lives of entities that look like humans, at the top. The problem of human-like robots is not purely abstract. There are examples of such robots, such as the robotic copy of Hiroshi Ishiguro or Sophia the robot.

Returning to the research of Nijssen et al. (2019), their dilemmas were structured in the same logic: “A group of people is in danger of dying or getting seriously injured, but they can be saved if the participant decides to perform an action that would mean sacrificing an individual agent (human, human-like robot, or machine-like robot) who would otherwise remain unharmed” (Nijssen et al., 2019, 45–46). From the perspective of the crime of failure to render aid, in every case people should sacrifice robots, and, if someone hesitated to do so in real life, they would be committing a crime.

In conclusion, robots with a human-like appearance are problematic from the perspective of the hierarchy of values embedded in legal systems. The law places the value of human life at the top of protected values. The lives of both animals and robots are worth less. In a conflict situation, we are obliged to save humans and sacrifice other entities, including robots. However, two problematic cases are possible: first, if people hesitate to sacrifice a robot, knowing that it is a robot, they commit a crime, and, second, if they hesitate to sacrifice a robot, thinking that it is a human, they do not commit a crime, but the consequence of their action (i.e., the human not being rescued) is undesirable in the legal system.

## Recommendations

In this section, I consider the appropriate response to the fact that human-like robots could pose a danger to human life by leading people to prioritize robot life. This prioritization could be done knowingly, if a person hesitates to sacrifice robots (e.g., due to empathy toward them) or unknowingly, if a person thinks that they are prioritizing a human that is in fact a robot. The deliberation in this case is based on the assumption that we want to preserve the contemporary hierarchy of values, in which human life is at the top of the protected entities in our legal system. Bryson used the term “human-centered society” (in contrast to “artifact-centered society”) (Bryson 2018). She recognized the dangers of over-attachment to robots and contended that we should respond to such dangers through design:

We design, manufacture, own and operate robots. They are entirely our responsibility. We determine their goals and behaviour, either directly or indirectly through specifying their intelligence, or even more indirectly by specifying how they acquire their own intelligence. But at the end of every indirection lies the fact that there would be no robots on this planet if it weren’t for deliberate human decisions to create them. (Bryson 2010, 65).

Bryson thus concluded that if there is a problem with the design of robots, we should change it in a way that will not cause unnecessary societal costs. In her other work, she formulated associated recommendations:

First, robots should not have deceptive appearance—they should not fool people into thinking they are similar to empathy-deserving moral patients. Second, their AI workings should be “transparent” […] The goal is that most healthy adult citizens should be able to make correctly-informed decisions about emotional and financial investment. As with fictional characters and plush toys […] we should be able to both experience beneficial emotional engagement, and to maintain explicit knowledge of an artefact’s lack of moral subjectivity. (Bryson 2018, 23)

Such recommendations could, in theory, provide a response to the issues discussed in this paper; however, the hope that robots will not be created to look like humans is unrealistic. Danaher, in response to such recommendations, observed that “[…] the drive to create robots that cross the performative threshold […] will probably prove too overwhelming for any system of norms (legal or moral) to constrain” (Danaher 2020, 2046). Gunkel also commented on Bryson’s recommendations by suggesting that thinking requires aestheticism, which should concern designers and users, and which he doubted is possible to enforce (Gunkel 2018a, 94). The desire to create entities that mirror humans is too strong to impose a general ban on creating robots in our own image, especially taking into consideration that robots with a human appearance are not unequivocally bad. There are dozens of areas of life in which robots that resemble humans would be beneficial, including sex robots or companion robots (cf. Di Nucci 2017; McArthur 2017; Ryland 2021). The fact that a knife can be used to commit a crime does not mean that we should ban the production of knives; they are too useful in everyday life. The same consideration applies to robots. We should minimize the potential negative outcomes from the existence of robots that mimic life rather than ban their creation, which seems to be neither possible nor sufficiently justifiable.

We should construct the world that we share with robots with consideration to how humans are. Humans tend to anthropomorphize objects: “Robots are now available in physical forms and can exhibit movements that are getting impressively more human. As a result, our brain, which has evolved to interact and understand humans, is tricked into interpreting their behavior as if it were generated by a human” (Sandini and Sciutti 2018, 7:1). Humans should take this tendency into account when discussing how to organize human–robot interactions. With regard to this topic, I offer two recommendations.

1. Humanoid robots should be easily distinguishable from humans.

People should know that they are interacting with robots. A person should be able to perceive that a robot is a robot at first glance. The fact that a robot is a robot should not be revealed only through interactions, but should also be evident from a distance. For example, robots should be easy to distinguish by drivers of cars for safety reasons, so they can be sure as to who should be sacrificed in a dilemmatic situation such as a car crash. Robots’ differences from humans should be apparent to help humans make appropriate decisions in dynamic situations requiring immediate reaction. This distinction may be achieved by incorporating a particular marking element into the design of robots, such as a light or an object protruding from the head.

This recommendation could be limited to certain robots used in certain contexts—especially where there is a threat to the safety of human beings. One example would be when robots go outside of the owner’s home and become a participant in traffic by crossing the street. This recommendation is comparable to the requirement that drones must not fly into certain zones, such as airports’ surroundings (cf. O’Malley 2019). There are reasons that justify the limitation of usage of technologies and adaptations to prioritize safety over other features, such as the freedom of flying whatever we like or to have a robot that looks a certain way, especially if the look becomes problematic. There is no need to have such limiting features for, for example, sex robots, which are and will be used almost exclusively in an intimate environment. Forcing the producer to make them look not human-like could even destroy the experience of using such robots.

2. Robots should inform other elements of the interactive environment that they are robots.

Robots should also inform other environmental elements that they are robots, even if the robots resemble humans. This will be essential in the context of autonomous cars, among other issues. There is an ongoing discussion around the ideal infrastructure of autonomous and connected vehicles (cf. Bonnefon et al., 2020), as well as what crash algorithms should be developed or implemented (cf. Nyholm 2018). From the perspective of the assumptions made in this paper, it is clear that humans should be saved; however, a car must know that something that resembles a human is not necessarily human. Cars that will replace human drivers are in development, and robots must inform such cars that they are not humans–not only through their appearance, but also in some way that may not be perceptible to humans.

It is possible that, in some cases, robots and other elements of the digital environment will “know” that robots that look like humans are not humans even without the implementation of this recommendation. The technological environment may progress beyond our current epistemological limitations and, for example, use temperature sensors that could differentiate human from non-human. We currently base our evaluations of objects, at least from a distance, mostly on visual aspects. If something looks like a human, we have no apparatus to determine that it is not a human. However, there are features that could help recognize humans among humanoid robots. One is body temperature, which is not visible to humans, but could be visible through technology. Nevertheless, if there is no temperature sensor in the future, or if that sensor will be insufficient to distinguish humans from humanoid robots in particular cases, then the proposed recommendation could be necessary.

The aim of this recommendation could be partially achieved in another way. We should make sure that the ways we are “teaching” technologies to recognize humans as elements of the environment are based not only on appearance. In cases of human-like robots, which will be elements of our social life, relying on the visual aspect could be misleading.

The proposed recommendations will not solve all of the problems caused by the deceptive human appearance of robots; rather, humans must decide based on real data. We should also communicate to society that, for now, human life has a unique value that is protected and requires other entities to be sacrificed to save it. Simply put, we should sacrifice robots to save humans, no matter how cute and human-like those robots may be.

## Conclusion

While the issue of robot rights may be unthinkable for some, it is nevertheless becoming an increasingly serious topic of scientific deliberation, and it is increasingly difficult to pretend that this topic is unimportant. The pressing factor is the number and sophistication of contemporary robots that increasingly resemble humans. Many issues must be resolved as soon as possible, including questions concerning how humans should treat robots. Claims that robots are mere property and should be treated as such are unsatisfactory, as our interactions–in both the research environment and in real life–demonstrate that people treat robots differently. Human relations with robots are intertwined with ethics and law.

In this paper, I have focused on the limits of the protection of robots, as illustrated by the moral dilemma of who should be saved between a human or a robot. I have discussed the issue from the perspective of various approaches of ascribing moral standing to robots and have demonstrated that prioritizing humans over robots may not always be the obvious course of action. I also explored the legal perspective, which protects the superiority of human beings as a manifestation of the hierarchy of values in legal systems. If we wish to preserve that hierarchy, we must react to the process of robots becoming more human-like. Our tendency to anthropomorphize robots could disrupt that hierarchy; in response, I have proposed recommendations that could be implemented at the level of robot design. Contemporary law is not fully ready for the coexistence of humans and human-like robots.

## Data Availability

The original contributions presented in the study are included in the article/supplementary material, further inquiries can be directed to the corresponding author.
